# Finger-Temperature-Detecting Liquid Crystal Composite Film for Anti-Counterfeiting Labels

**DOI:** 10.3390/molecules25030521

**Published:** 2020-01-25

**Authors:** Yanzi Gao, Ke Feng, Jin Zhang, Lanying Zhang

**Affiliations:** 1 School of Economics, Peking University, Beijing 100871, China; gaoyanzi@pku.edu.cn (Y.G.); zhangjin_ada@pku.edu.cn (J.Z.); 2 Department of Materials Science and Engineering, College of Engineering, Peking University, Beijing 100871, China.; zhanglanying@pku.edu.cn; 3 School of Software and Microelectronics, Peking University, Beijing 100871, China

**Keywords:** liquid crystal, anti-fake, cholesteric phase, side-chain liquid crystal polymers

## Abstract

The development of the economy has increased the demand for anti-counterfeiting technologies, and with the traditional ones becoming known to the public, new and more effective ones are needed. In this study, a series of liquid crystal mixtures containing side-chain liquid crystal polymers and small-molecular-weight liquid crystals (LCs) were designed and prepared. The phase transition behavior and self-assembling structures of the LC mixtures were investigated by a combination of differential scanning calorimetry, polarized optical microscopy, and small-angle X-ray diffraction. The optical properties of the mixture film were characterized with a UV/VIS/IR spectrum study. The results reveal that the obtained film exhibited different optical modes between transparent, scattering, and selective reflection under finger-temperature control. Therefore, by the introduction of a coexisting thermal- or optical-polymer-dispersed network, a liquid crystal composite film with an integration of apparent optical switching modes and enhanced strength and toughness was successfully demonstrated. This research provides a versatile strategy for the design and preparation of liquid crystal anti-counterfeiting materials for practical use. In this study, a prototype finger-temperature-detecting anti-counterfeiting label was prepared, and its temperature-response property was demonstrated.

## 1. Introduction

Due to the fast development of the global economy during the last several decades, counterfeiting is creating more and more profit. Meanwhile, by utilizing continuously upgraded high-resolution scanners and printers, counterfeiters are manufacturing forgeries that are harder to distinguish, and they are stretching their diabolical claws to more profitable areas, such as bills and banknotes [1,2,3]. This not only violates the interests of the product manufacturers but also disrupts economic order. For instance, counterfeit banknotes and bank bills cause both domestic and global economic problems, and counterfeit labels help to cheat buyers and cause damage to the real producers.

For decades, anti-counterfeiting technologies have been developed to fight counterfeiting, such as holograms [4], watermarks [5], and coated labels [6], but these methods have also become known to the counterfeiters. Consequently, the development of the next generation of anti-counterfeiting materials and technologies is urgently needed. Recently, new anti-counterfeiting technologies have been invented to meet the demand. Generally, according to the anti-counterfeiting mechanism, anti-counterfeiting technologies can be divided into the following categories: fluorometric approaches [7,8], colorimetric approaches [9,10,11], organic electronic approaches [12,13], and mass-spectrometry encryption [14,15]. Zhu et al. developed a film-type security device utilizing a dual-responsive fluorescence switch comprising a naphthalimide dimer tethered to a photochromic bisthienylethene bridge [7]. Under sunlight, emissions from the fluorophore could be observed when the bisthienylethene moiety was in its open-ring form, while under UV irradiation, fluorescence quenching happened. Based on these properties, a security image could be fabricated using a bisthienylethene-bridge embedded polymethylmethacrylate (PMMA) film, which may find potential application in the field of security recording. By using the reversible structural change between two isomers, which takes place upon irradiation at an appropriate wavelength of photochromic materials, Kobatake et al. developed a secret display anti-fake material [16]. The label showed different patents at different irradiation states, which could be used for UV-detecting anti-counterfeiting labels. Zschieschang et al. described a carefully designed organic thin-film transistor (TFT) that was embedded in a banknote [12]. Using this polymer ferroelectric memory device, the fabrication of organic circuits on banknotes will become a promising approach for developing organic electronic-based anti-counterfeiting systems. Cooks et al. described a technique that employs selective mass imaging of a particular ink molecule in a document by desorption electrospray ionization mass spectrometry, which could identify the secondary scattered droplets [14].

Liquid crystal (LC), which exhibits excellent light-controllable characteristics and a fast response to external stimuli, such as light, electricity, magnets, temperature, and pressure, is regarded as one of the most promising anti-counterfeiting materials and has recently sparked enormous interest in scientists. K. Nakayama and J. Ohtsubo proposed an optical security device using the fingerprint texture in the N* phase, which has both the function of a fingerprint for authenticity and the function of storage for a designed pattern [17]. The stored pattern is visible to the naked eye without using polarizers, and such devices have the potential to be used for anti-counterfeiting purposes. By embedding periodically arranged squares with a planar and a vertical texture into the background with a developable-modulation-type fingerprint texture using a two-step photo-polymerization technique, W. S. Li et al. realized two types of checker-patterned polymer-stabilized cholesteric LC textures [18]. A graphic symbol with a miniaturized 2D barcode pattern that combines dark squares with vertical textures and a light background with a fingerprint texture was demonstrated, and these enhanced anti-counterfeiting features are difficult to falsify or duplicate. K. Nakayama and J. Ohtsubo quantitatively examined schlieren textures in the nematic (N) phase, which is simpler than quantitatively examining fingerprint textures in the N* phase, for applications to security devices using cross correlation [19]. The minimum correlation coefficients of the written patterns were 0.96, which indicates that the written patterns using the same photomask were almost the same. Furthermore, the patterns of the schlieren textures in different cells were also evaluated, and the maximum coefficients were 0.09, indicating that their patterns in different cells were unique. These results suggest that the non-uniform patterns of schlieren textures have potential applications in anti-counterfeiting materials.

In this paper, by the simple mixing of a cholesteric side-chain liquid crystal polymer (ChSCLCP), a smectic side-chain liquid crystal polymer (SmSCLCP), the nematic LC SLC1717, and the chiral dopant S811 with different proportions, a series of LC films with artificially adjustable phase transition behaviors were successfully prepared. Interestingly, the LC film can exhibit different optical modes between transparent, scattering, and selective reflection at finger temperature. Furthermore, by the introduction of a polymer-dispersed liquid crystal (PDLC) network based on urethane acrylate and isophorone diisocyanate/tetraethylene glycol, a LC composite film with an integration of apparent optical switching modes and enhanced strength and toughness was obtained. The resultant composite film is therefore a model system for anti-counterfeiting labels. We strongly believe that it could offer an innovative insight into the design and preparation of LC anti-counterfeiting materials for practical use.

## 2. Results and Discussion

### 2.1. Phase Behaviors and Phase Structures of the LC Mixtures

**Scheme 1:**
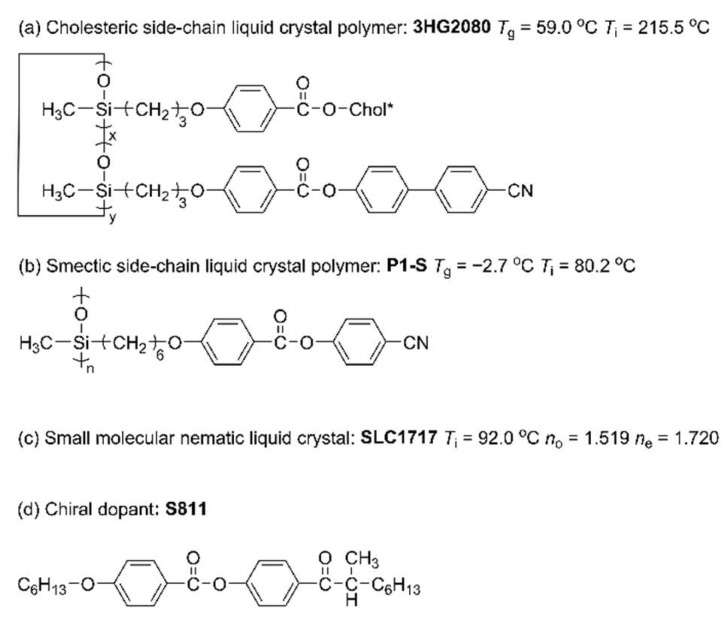
The chemical structures of the materials used in this work.

The LC materials used in this work are listed in Scheme 1. The ChSCLCP and SmSCLCP were obtained via the conventional free-radical polymerization of different liquid crystalline monomers, and details of the synthetic route are referred to in our group’s previous work [20,21]. In the present experiment, by rationally adjusting the proportion of the ChSCLCP, several LC mixtures with different phase transition behaviors were obtained, as shown in Table 1. The compositions, glass transition temperature, and the phase transition temperatures of the samples are also listed in the table.

**Figure 1:**
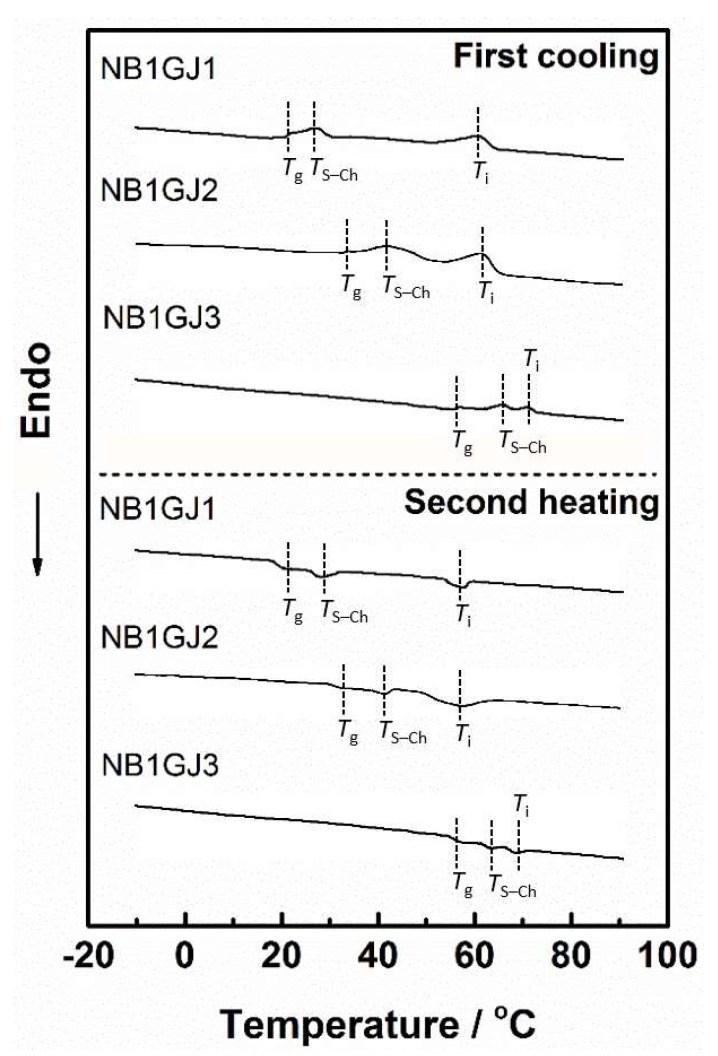
Differential scanning calorimetry curves of the samples during the first cooling and second heating scans at a rate of 20 °C min^−1^.

The phase transition behaviors and phase structures of all the LC mixtures were examined by a combination of DSC, POM, and small-angle X-ray scattering (SAXS) measurements. As shown in Figure 1, three endothermic/exothermic peaks, which may be related to the glass transition (T_g_), LC phase transition, and the isotropic transition from a possible LC phase, were detected in each of the DSC curves for all of the samples during the first cooling and second heating processes. Both the T_g_ and the clearing point temperature (T_i_) gradually increased with the increasing content of the ChSCLCP, while the mesogenic range was narrowed. Furthermore, it is worth noting that the phase transition temperatures of NB1GJ1 were all around finger temperature, which makes its use possible for interesting applications.

**Figure 2:**
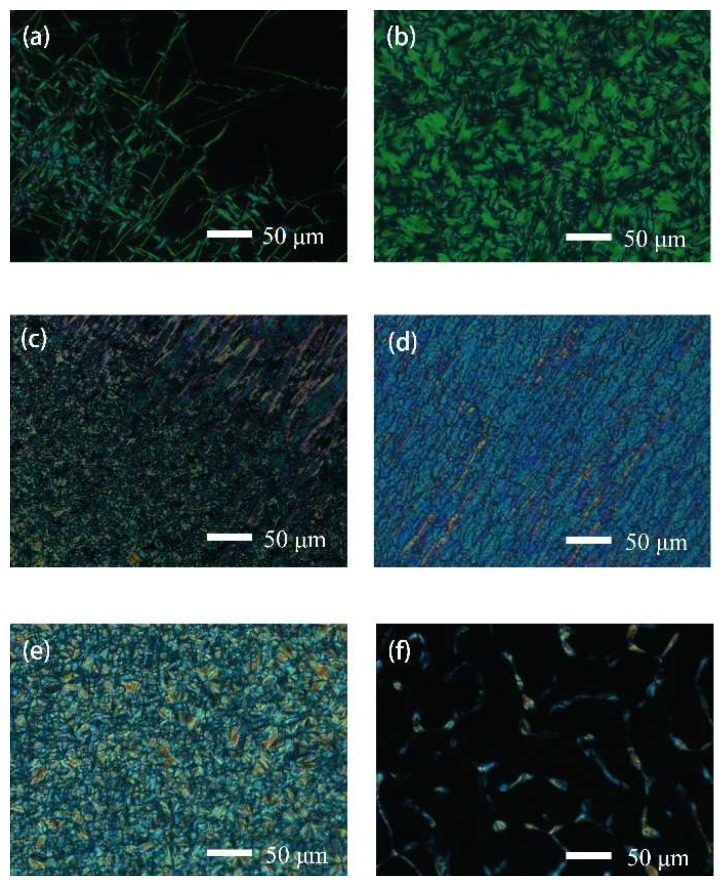
Typical polarized optical microscopic images of NB1GJ1 at different temperatures heated from glass states: (**a**) 21.2 °C, (**b**) 25.0 °C, (**c**) 29.2 °C, (**d**) 36.5 °C, (**e**) 58.4 °C, and (**f**) 58.6 °C.

To further confirm the phase transition behaviors and phase structures of all the samples, POM and SAXS techniques were utilized. The samples were filled into the cells, whose inner surfaces had been planar orientated, by capillary action in order to perform POM observation and were sandwiched between aluminum films for the SAXS experiment. Figure 2 shows the typical LC textures of NB1GJ1 at different temperatures during the heating process. Upon heating to near ambient temperature (as shown in Figure 2a,b), the LC mixture developed a representative fan-shaped texture, indicating that the LC phase may have been a typical smectic phase. Upon further heating to over 29 °C (as shown in Figure 2c,d), the fan-shaped texture gradually changed to the oily streak texture, a characteristic planar state of a cholesteric (Ch) phase. When the temperature was near the clearing point, the oily streak morphology slowly transformed to the focal conic texture (as shown in Figure 2e), another typical texture of a Ch phase, then disappeared slowly after the isotropic temperatures (as shown in Figure 2f). Accordingly, while cooling from the isotropic state, the focal conic and oily streak textures, which are characteristics of a Ch phase, developed again. Upon further cooling to temperatures below the phase transition temperature, the oily streak textures transformed to a fan texture and then the whole field of view became black again.

**Figure 3:**
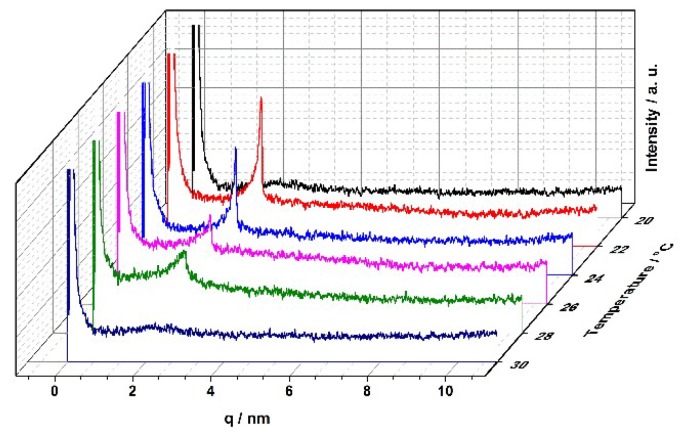
Small-angle X-ray scattering (SAXS) patterns of NB1GJ1 at different temperatures during the heating process.

Figure 3 shows the SAXS patterns of the NB1GJ1 sample at different temperatures during the heating process. The very sharp diffraction peaks in the low-angle region at low temperatures suggested a smectic phase structure. Upon heating, the intensity of the diffraction peaks dramatically decreased and became a scattering halo when the temperature reached 28 °C, indicating that a phase transition from smectic to cholesteric had happened, which was in accordance with the results of the DSC and POM experiments.

By combining the results of the DSC, POM, and SAXS experiments, the self-assembly behaviors of the LC mixture NB1GJ1 can be concluded as follows: a simple smectic phase, a Ch phase, and an isotropic state. Similarly, the phase transition behaviors and phase structures of other LC mixtures were also investigated, and the phase transition temperatures could be artificially tuned by changing the content of the ChSCLCP.

### 2.2. Optical Properties of the LC Mixture Film

**Figure 4:**
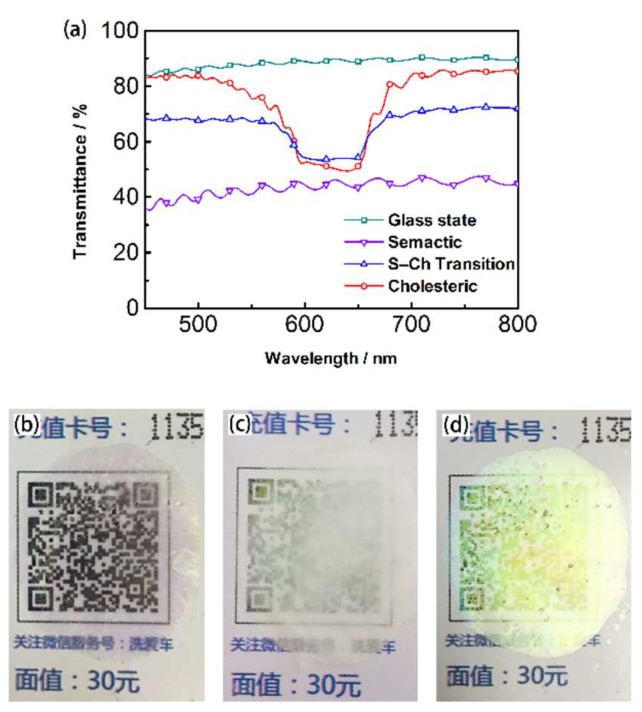
(**a**) The transmission spectra of the samples at different temperatures. Pictures of the liquid crystal (LC) film during the finger heating process: (**b**) transparent state, (**c**) scattering state, (**d**) both reflection and scattering states, and (**e**) reflection state.

Due to the abundant LC phase structures with the tunable phase transition temperatures near room temperature, we speculated that there may be interesting optical properties under temperature stimulation. The LC mixture NB1GJ1 was filled into the cells by capillary action to prepare the LC film, and a variable temperature UV/VIS/IR spectrum study was conducted, as shown in Figure 4a. Corresponding to the phase transition from the glass state to the smectic phase, the transmittance of the LC film was very high in the glass state, however, it decreased to below 40% in the smectic phase, which indicated that the LC film changed from a transparent to a scattering state. With the transformation from the smectic phase to the Ch phase and the formation of a planar texture of the Ch state, the LC film acquired a selective reflection mode with transmittance of about 70% beyond the reflection wavelength. When the development of the Ch phase was complete, the transmittance of the film increased to about 80% beyond the reflection wavelength. Upon further heating to above the clearing point, the planar texture was destroyed and the LC film became transparent. To visually observe the optical behavior changes of the film more clearly, we put the film over a quick response (QR) code and stimulated it by continuous finger touch. Interestingly, as shown in Figure 4b,c,d,e, we saw a gradual change of the LC film images at body temperature within seconds. The film was in transparent state at room temperature (Figure 4b); after being heated by a finger for 3 s, the film changed to a scattering state (as shown in Figure 4c). After further touching for 1 s, due to the development of a planar texture induced by the side-chain liquid crystal polymer (SCLCP) [22], the LC film showed both reflection and scattering states (Figure 4d). After further touching for 2 s, the planar texture developed completely and the LC film selectively reflected green light (Figure 4e). Overall, the LC film could readily perform four optical states (transparent, scattering, both reflection and scattering, and reflection) through body-temperature regulation, which indicated that the LC material may be an ideal anti-counterfeiting material for practical application.

**Figure 5:**
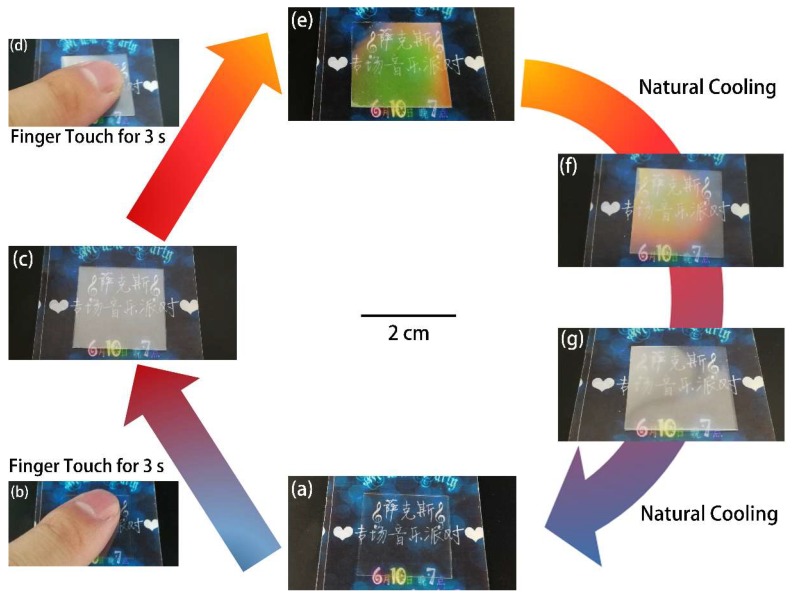
Pictures of the LC anti-counterfeiting label under finger touch: (**a**) transparent state at room temperature, (**b**) continuous finger touch for 3 s, (**c**) scattering state with smectic phase, (**d**) continuous finger touch for further 3 s, (**e**) reflection state with Ch phase, (**f**) both reflection and scattering states, and (**g**) scattering state with smectic phase during natural cooling.

Figure 5 shows a device application of the finger-temperature-responsive LC composite film for anti-counterfeiting labels.

**Figure 6:**
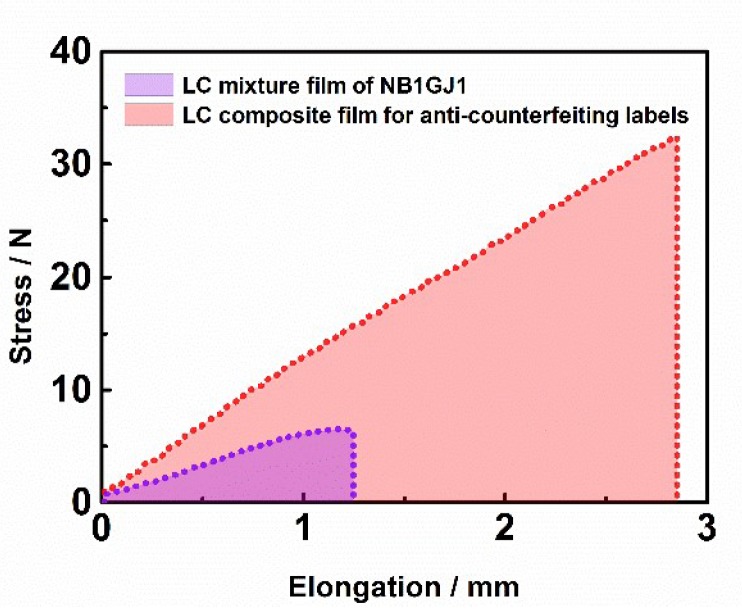
Peeling-strength results of the corresponding samples.

According to the results described above, the LC mixture film of NB1GJ1 exhibited abundant optical modes under body-temperature regulation, which could be favorable for application in the intelligent anti-counterfeiting field. Thus, a LC composite film for practical anti-counterfeiting label use was prepared by introducing a coexisting polymer-dispersed network of a photo-polymerization network based on urethane acrylate and a thermal-polymerization network based on isophorone diisocyanate/tetraethylene glycol. Here the coexisting polymer-dispersed network played two roles: (1) the photo-polymer-dispersed network mainly increased the toughness while anchoring the planar texture of cholesteric LC and (2) the thermal-polymer-dispersed network further enhanced the peeling strength of the film. More importantly, the additional polymer-dispersed networks had no effect on the optical properties of the obtained composite film. Figure 5 shows the pictures of the LC composite anti-counterfeiting label under finger touch. The label was transparent at ambient temperature (Figure 5a). After finger touching for 3 s, the LC mixture was at a smectic state, and accordingly, the LC composite anti-fake label showed a scattering state (Figure 5c). Upon further finger touching for 3 s, the LC mixture developed the planar texture of the cholesteric phase and the LC composite anti-fake label displayed a reflection state (Figure 5e). During the natural cooling process, the label first exhibited both reflection and scattering states (Figure 5f), then showed a scattering state (Figure 5g), and finally became transparent (Figure 5a). Additionally, the mechanical behavior of the composite film was also tested. As shown in Figure 6, the largest peeling strength of the LC composite film for anti-counterfeiting labels was nearly six times more than that of the LC mixture film of NB1GJ1, proving that the introduction of a polymer network greatly enhanced the peeling strength of the film. Furthermore, the maximum elongation of the LC composite film for anti-counterfeiting labels was nearly two times of that of the LC mixture film of NB1GJ1, indicating that the polymer network sufficiently improved the toughness of the film. These results, combined with the results of the mechanical property tests, show that the film exhibited a relatively excellent comprehensive performance and was proved to have a practical application in anti-counterfeiting labels.

## 3. Experimental Section

### 3.1. Materials

The nematic LC SLC1717 (T_Cr-N_ < –40.0 °C, T_N-I_ = 91.8 °C) was purchased from Shijiazhuang Yongsheng Huatsing Liquid Crystal Co., Ltd. (Shijiazhuang City, Hebei Province, China). Poly(3-mercaptopropylmethylsiloxane) (PMMS, SMS-992, M.W. 4000–7000, 95 cst, Gelest Inc., Morrisville, NC, USA), ethylparaben (analytically pure grade, Sinopharm, Beijing, China), cholesterol (analytically pure grade, Sinopharm, Beijing, China), 3-bromopropene (98%, Beijing Dominant Technology Co., Beijing, China), dimethylaminopyridine (DMAP) (99%, Energy Chemical, Beijing, China), N,N0-dicyclohexylcarbodiimide (DCC) (98%, Energy Chemical, Beijing, China), potassium hydroxide (analytically pure grade, Beijing Chemical Reagents Co., Beijing, China), S811 (Jiangsu Hecheng Display Technology Co., Ltd, Nanjing City, Jiangsu Province, China), and anhydrous potassium carbonate (analytically pure grade, Beijing Chemical Reagents Co., Beijing, China) were commercially available and used without any further purification. Toluene (analytically pure grade, Beijing Chemical Reagents Co., Beijing, China) was refluxed over sodium and distilled under nitrogen atmosphere. Azodiisobutyronitrile (AIBN) (analytically pure grade, Beijing Dominant Technology Co., Beijing, China) was purified by recrystallization from ethanol. The other chemical reagents were used as received.

### 3.2. Measurements

All ^1^H-NMR spectra of the samples were collected on a Bruker HW400 MHz spectrometer (ADVANCE III-400, Bruker Corporation, Coventry, UK) using deuterated chloroform (CDCl_3_) or dimethylsulfoxide (DMSO) with tetramethylsilane (TMS) as the internal standard at room temperature. The FTIR spectra were recorded using a PerkinElmer spectrum 100 spectrophotometer (PerkinElmer, Waltham, Massachusetts, USA). A PerkinElmer DSC8000 (PerkinElmer, Waltham, MA, USA) with a mechanical refrigerator was used to obtain the differential scanning calorimetry (DSC) results and the phase transitions of the polymers under dry nitrogen at a heating and a cooling rate of 20 °C min^−1^. The temperature and heat-flow scale were calibrated using zinc and indium as standard. Polarized optical microscopy (POM) was carried out on a Carl Zeiss Axio Vision SE64 (Carl Zeiss AG, Jena, Germany) polarized optical microscope with a Linkam LTS420 hot stage (Linkam, London, UK). Spectral characterization was done by an unpolarized UV/VIS/IR spectrophotometer (PerkinElmer Lambda 950, PerkinElmer, Waltham, MA, USA) in transmission mode at normal incidence. The peeling-strength experiment was practiced on a universal tensile test machine (Instron 5969, Instron, Norwood, MA, USA).

### 3.3. Preparation of the LC Composite Film for Anti-Fake Labels

The LC cells were manufactured with two glass substrates, and polyethylene terephthalate (PET) films of 20 μm thickness were used as cell spacers. To prepare the LC film samples, capillary action was used to fill the cells.

A mixture of NB1GJ1 (prepared LC sample), urethane acrylate (photo-polymerizable monomer), isocyanate (thermal-polymerizable monomer), a photoinitiator (Irgacure 651), and a thermal initiator (Dibutyltin Dilaurate) was prepared homogeneously in certain proportions. Then the mixture was used to fill between two layers of PET film with a thickness of 20 ± 1 μm controlled by a spacer. After this, the mixture was irradiated by a UV lamp (365 nm 35 W Hg lamp, PS135, UV Flood, Stockholm, Sweden) for 30 min to introduce the photo-polymer-dispersed network and then heated in an oven at 363.15 K for 7 h to introduce the thermal-polymer-dispersed network.

## 4. Conclusions

In summary, based on the SCLCPs/small-molecular-weight LC mixtures and the coexisting polymer-dispersed networks, a finger-temperature-detecting LC composite film for anti-counterfeiting labels was prepared with the following advantages: easily observed multi-state switchable optical modes, responsive to finger touch; enhanced mechanical properties without the need for a substrate orientation process; and an artificial tunable response temperature. Experiments to further improve the mechanical performance of the composite films for practical application in the anti-counterfeiting field are under way.

## Figures and Tables

**Table 1:** The compositions and phase transitions of all the samples.

Sample	SLC1717/S811/P1-S/3HG2080 [weight%]	*T*_g_^a^ [°C]	*T*_S‒Ch _^a^ [°C]	*T*_i_^a^ [°C]
NB1GJ1	53.80/24.40/15.10/6.70	21.12	29.17	58.48
NB1GJ2	50.42/22.87/14.15/12.56	33.52	39.37	61.81
NB1GJ3	36.62/16.61/10.28/36.49	57.60	66.84	72.42

^a^ The glass transition temperatures (*T*_g_), phase transition temperature between smectic and cholesteric (*T*_S‒Ch_), and clearing temperatures (*T*_i_) were determined by differential scanning calorimetry (DSC)during the first cooling scan under nitrogen at a rate of 20 °C min^−1^.
